# Knock out hepatic Krüppel-like factor 16 (KLF16) improve myocardial damage and promoted myocardial protection of myocardial ischemia-reperfusion via anti-oxidative and anti-inflammation effects by TFAM/PPARβ signal passage

**DOI:** 10.1080/21655979.2021.1982302

**Published:** 2021-11-25

**Authors:** Yue Xin, Pixiong Su, Yan Liu, Song Gu, Xiangguang an, Xitao Zhang, Jun Yan, Yulin Guo, Jian Zhou, Guilin Yang

**Affiliations:** Department of Cardiac Surgery, Beijing Chao-Yang Hospital, Capital Medical University, Beijing, China

**Keywords:** KLF16, TFAM, myocardial ischemia-reperfusion

## Abstract

This study is aimed at investigating mechanisms and effects of Krüppel-like factor 16 (KLF16) affects myocardial ischemia-reperfusion. Patients with myocardial ischemia-reperfusion and normal volunteer were collected. C57BL6J male mice were located left anterior descending coronary artery (LAD). H9c2 cell was induced with hydrogen peroxide (H2O2) and Lipopolysaccharide (LPS). Serum KLF16 mRNA expression was increased in myocardial ischemia-reperfusion. Serum mRNA of KLF16 was positive correlation with serum creatine kinase MB (CK-MB) or creatine kinase (CK) levels in patients with myocardial ischemia-reperfusion. The expression of KLF16 mRNA and protein in mice with myocardial ischemia-reperfusion were also increased. The inhibition of KLF16 reduced oxidative stress and inflammation, and presented myocardial ischemia (MI) in vivo model of myocardial ischemia-reperfusion. Mitochondrial transcription factor A (**TFAM**)/peroxisome proliferator-activated receptor-beta (PPARβ) signal passage is target spot of KLF16 in Myocardial ischemia-reperfusion. TFAM interlink KLF16 in myocardial ischemia-reperfusion. TFAM participate in KLF16 affects myocardial ischemia-reperfusion. PPARβ promoter region KLF16 affects myocardial ischemia-reperfusion. These results firstly demonstrated that knock-out KLF16 reduced oxidative stress and inflammation, and presented MI in vivo model of myocardial ischemia-reperfusion through the induction of PPARβ by TFAM, may provide a novel therapeutic strategy for myocardial ischemia-reperfusion.

## Introduction

Under normal circumstances, the onset of inflammation is relatively fast, which persists during the process of heart failure (HF) after myocardial infarction (MI) [1]. A certain degree of inflammation can alleviate the symptoms of MI and promote the body to restore the blood in myocardium [2]. However, the excessive inflammatory response can further aggravate the symptoms of HF after MI [3].

Oxidative stress can not only attack cell membranes and the organelles but can also induce the occurrence of inflammatory responses through the mutual enhancement of inflammatory factors, further aggravating myocardial damage caused by MI [3]. Therefore, increasing the level of antioxidant enzymes and decreasing the content of ROS are generally considered as one of the important therapeutic strategies for MI [3]. MI can increase the level of oxidative stress in myocardial tissue to further cause oxidative damage in myocardial tissue [3]. As a type of second messenger, reactive oxygen species (ROS) can transmit stress signals and exert various effects on cells [3]. However, excessive ROS can cause cell death or necrosis. It is widely recognized that MI can cause oxidative stress, leading to pathological myocardial remodeling and myocardial hypertrophy [3].

Transcription factor A (TFAM) is a key regulator of mitochondrial DNA replication, decreased reactive oxygen species (ROS), increased mitochondrial fuel oxidation, and protected against IR [4]. PPARβ act as ligand-inducible transcription factors, demonstrate pleiotropic cardioprotective properties and exhibit antioxidant and anti-inflammatory properties [5].

Krüppel-like transcription factor 16 (KLF16) is a member of the Krüppel-like transcription factor family and is involved in the cell cycle and promoter-dependent transcriptional regulation [3]. KLF16 plays a tumor suppressor role in lung adenocarcinoma by inhibiting tumor cell proliferation and inducing apoptosis [3]. This study is aimed at investigating mechanisms and effects of KLF16 affects myocardial ischemia-reperfusion.

## Materials and methods

### Clinical samples

Patients with myocardial ischemia-reperfusion (n = 12) and normal volunteer (n = 12) were collected from Beijing Chao-Yang Hospital, Capital Medical University. All the samples were obtained following patient consent and approval by the Ethics Committee of Beijing Chao-Yang Hospital, Capital Medical University. Serum samples were collected and saved at −80°C. Written informed consent was obtained from each patient before samples were taken. All Patients with myocardial ischemia-reperfusion was required for enrollment: a TIMI (Thrombolysis In Myocardial Infarction) risk score ≥3. Exclusion criteria: atrial fibrillation, bundle branch block, left ventricular hypertrophy, history of prior hemorrhagic stroke.

### Vivo animals model

C57BL6J male mice were used in this experiment procedure and received ordinary feed and had free access to food and water for 1 week. All experimental procedures involving animals were approved by Institutional Animal Care and Use Committee of Beijing Chao-Yang Hospital, Capital Medical University. Mice was injected with 50 mg/kg pentobarbital sodium and fixed on the operating table. Then, heart was exposed, pericardium was removed, and left anterior descending coronary artery (LAD) was located. The ligation point changed from red to white, and the intraoperative electrocardiogram displayed the ST segment after ligated with myocardium for MI model as document [6]. Anti-KLF16 group, mice were intraperitoneally injected with 200 pg/3 days of anti-KLF16 body for 4 weeks. KLF16 group, mice were intraperitoneally injected with 1 μg/3 days of KLF16 human recombinant protein for 4 weeks.

### Histopathologic assay

The heart tissues were fixed with 4% paraformaldehyde for 24 h. Tissue samples were embedded in paraffin, and the 5-μm -thick sections were stained with hematoxylineosin (H&E). Tissue samples were obtained using fluorescence microscopy (Nikon Eclipse TE2000-U, Japan).

### Microarray analysis

Total RNA was extracted from serum samples, and the amount of RNA was quantified by use of NanoDrop 1000. Total RNA of each sample was used for reverse transcription using an Invitrogen SuperScript double stranded cDNA synthesis kit. Double-stranded cDNA was executed with a NimbleGen one-color DNA labeling kit and then executed for array hybridization using NimbleGen hybridization system and washing with the NimbleGen wash buffer kit. Axon GenePix 4000B microarray scanner (Molecular Devices) was used for scanning.

### Quantitative RT-PCR (Q-PCR) analysis

Total RNA was isolated with TRIzol (Invitrogen) and total RNA (200 ng) was used to synthesize cDNA using a ReverTra Ace qPCR RT Kit (Toyobo, Tokyo, Japan). Q-PCR was performed with SYBR green detection using ABI 7900 real-time PCR system (Promega). The expression levels were analyzed using the threshold cycle (Ct) method.

### Cell culture and transfection

Cardiac muscle cell H9c2 cell lines were obtained from Shanghai Cell Bank, Chinese Academy of Sciences, and cultured in Dulbecco’s modified Eagle’s medium (DMEM, Invitrogen, Carlsbad, CA, USA) which was supplemented with 10% fetal bovine serum (FBS, Invitrogen, Carlsbad, CA, USA) in a 5% CO2 and 95% air.

2.5 μg of plasmid DNA (KLF16 or TFAM) or 200 ng of si-mimics (S KLF16 or TFAM) and 5 μL of Lipofectamine 2000 (Invitrogen, Carlsbad, CA, USA) were diluted with 250 μL Opti-MEM, respectively, and incubated for 5 min at room temperature. After 48 h of transfection, cell was induced with 250 μM hydrogen peroxide (H2O2) and 100 ng of Lipopolysaccharide (LPS) for 24 h as document [6,7].

### Enzyme-Linked Immunosorbent Assay (ELISA) kit for inflammation

CK (A032-1-1), CK-MB (H197-1-1), IL-1β (H002), IL-6 (H007-1-1), TNF-α (H052-1), INF-γ (H052-1), ROS production (E004-1-1), MDA (A003-1-2), SOD (A001-3-2), GSH (A006-2-1) and GSH-PX (A005-1-2) levels in heart tissue or cultured supernatants were quantified using an ELISA kit according to the manufacturer’s instructions (Nanjing Jiancheng Bioengineering Research Institute).

### Luciferase reporter assay

HEK293T cells were used to measure luciferase reporter. After 48 h transfection with TFAM mimics, 500  ng pcDNA3.1 vector or pcDNA3.1- KLF16-WT, pcDNA3.1- KLF16-Mut, HEK293T cells were harvested for luciferase activity assessment using a dual-luciferase reporter assay system (Promega).

### Immunofluorescence

Cells were fixed with 4% paraformaldehyde for 15 min and incubated with 0.25% of TrisionX100 for 15 min at room temperature. Cells were blocked with 4% BSA in PBS for 1 h and then incubated with anti-TFAM (Abcam, Cambridge, MA, USA), anti-PPARβ (Abcam, Cambridge, MA, USA), anti-KLF16 (Abcam, Cambridge, MA, USA) at 4°C over-night. Nuclear were stained by DAPI and mitochondrion (Mito-Tracker Red CMXRos). The images of was obtained using a Zeiss Axioplan 2 fluorescent microscope (carl Zeiss AG, Oberkochen, Germany) and analysis was performed using Image-pro plus 6.0 (Media Cybernetics, Inc., Rockville, MD, USA) software.

### Western blot analysis

Protein extracts were prepared with RIPA buffer containing a protease inhibitor mixture and protein concentrations were determined using BCA Protein Assay. Proteins were transferred to the membrane and blocked with 5% skim milk for 1 h after being separated by sodium dodecyl sulfate-polyacrylamide gel electrophoresis (SDS-PAGE). The specific primary antibody: anti-TFAM (ab176558, 1:1000, Abcam, Cambridge, MA, USA), anti-PPARβ (ab178860, 1:1000, Abcam, Cambridge, MA, USA), anti-KLF16 (sc-377,519, 1:500, Santa Cruz Biotechnology) and anti-β-Actin (sc-8432, 1:5000, Santa Cruz Biotechnology, Santa Cruz, CA, USA) was used to incubate with the membrane overnight at 4°C. After being washed with tris buffered saline-tween (TBST) for 15 min, the secondary antibody (sc-2005, sc-2006, 1:5000, Santa Cruz Biotechnology, Santa Cruz, CA, USA) was used to incubate the membrane for 2 h at room temperature. Protein bands were visualized via enhanced chemiluminescence (Thermo Fisher, USA) and quantified using ImageJ software (version 1.48 v, NIH, Bethesda, MD).

### Statistical analysis

Data are expressed as mean ± S.E. The differences between the groups were analyzed by using the Student’s t-test or two-way ANOVA followed by a least significant distance post hoc test. a P < 0.05 was considered statistic significant.

## Results

### KLF16 expression levels in model of myocardial ischemia-reperfusion

**Figure 1.**
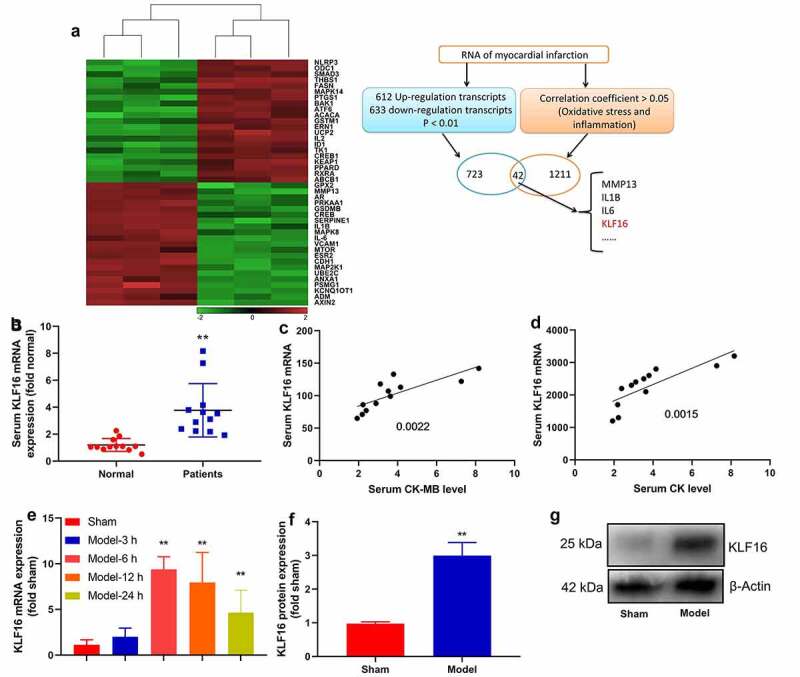
KLF16 expression levels in model of myocardial ischemia-reperfusion

First of all, this experiment explored the changes of regulatory gene in model of myocardial ischemia-reperfusion, we used microarray analysis to understandgene expression levels in model of myocardial ischemia-reperfusion (Figure 1(a)). Then these results of qRT-PCR revealed that serum KLF16 mRNA expression was more effectively increased in myocardial ischemia-reperfusion (Figure 1(b)). Serum mRNA of KLF16 was negative correlation with serum CK-MB or CK levels in patients with myocardial ischemia-reperfusion (Figures 1(c,d)). The expression of KLF16 mRNA in mice with myocardial ischemia-reperfusion was up-regulated at time dependence (Figure 1(e)). The expression of KLF16 protein in mice with myocardial ischemia-reperfusion was also increased (Figure 1(g)). In general, KLF16 is identified to be highly expressed in model of myocardial ischemia-reperfusion.

Heat map of gene chip (a), KLF16 mRNA expression levels in patients with MI model (b), serum mRNA of KLF16 was negative correlation with serum CK-MB or CK levels in patients with myocardial ischemia-reperfusion (c and d) (n = 12);KLF16 mRNA and protein expression in mice with myocardial ischemia-reperfusion (e, f and g) (n = 6). Normal, normal volunteer group; Patients, Patients with myocardial ischemia-reperfusion. Sham, sham control group; Model, MI mice group. **p < 0.01 compared with sham control group or normal volunteer group.

### KLF16 regulated oxidative stress and inflammation in vivo model of myocardial ischemia-reperfusion

**Figure 2.**
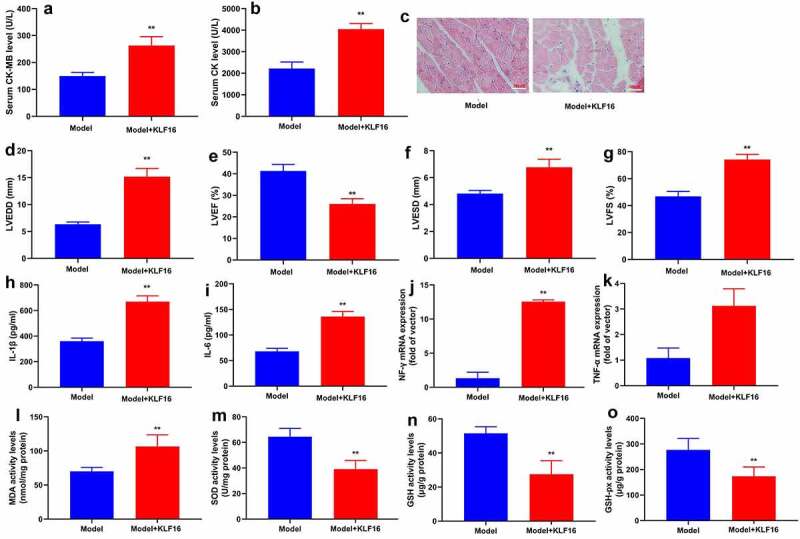
KLF16 regulated oxidative stress and inflammation in vivo model of myocardial ischemia-reperfusion

In order to elucidate the function of KLF16 in vivo and vitro models of myocardial ischemia-reperfusion, human KLF16 recombinant protein or anti-KLF16 body was injected into mice with myocardial ischemia-reperfusion. Human KLF16 recombinant protein decreased serum CK-MB and CK levels, inhibited myocardial damage, diminished LVEDD, LVESD and LVSD levels, and increased LVEF level in mice with myocardial ischemia-reperfusion (Figure 2(a-g)). Human KLF16 recombinant protein lessened MDA level, accelerated SOD, GSH and GSH-PX levels, and weakened IL-1β, IL-6, TNF-α and INF-γ levels in heart tissue of mice with myocardial ischemia-reperfusion (Figure 2(h-o)).

Serum of CK-MB and CK level (a and b), HE for myocardium fiber (c), left ventricular internal diameter (d), left ventricular ejection fraction (e), left ventricular fractional shortening (f) and Left ventricular stroke volume (g); MDA, SOD, GSH, GSH-PX levels in heart tissue (h, i, j and k); IL-1β, IL-6, TNF-α and INF-γ levels in heart tissue (l, m, n and o). Model, MI mice group; Model+KLF16, MI mice with KLF16 human recombinant protein group. n = 6; **p < 0.01 compared with MI mice group.

**Figure 3.**
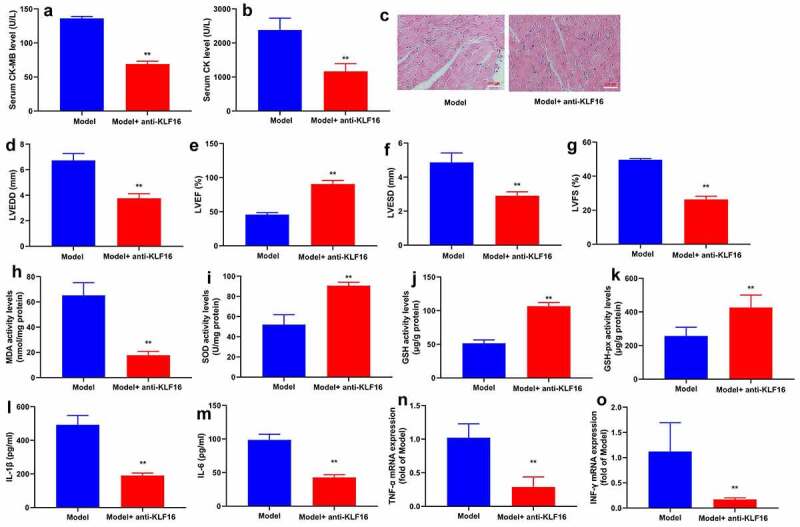
Anti-KLF16 regulated oxidative stress and inflammation in vivo model of myocardial ischemia-reperfusion

Anti-KLF16 body heightened serum CK-MB and CK levels, increased myocardial damage, induced LVEDD, LVESD and LVSD levels, and diminished LVEF level in mice with myocardial ischemia-reperfusion (Figure 3(a-g)). Anti-KLF16 body also accelerated MDA level, lessened SOD, GSH and GSH-PX levels, and promoted IL-1β, IL-6, TNF-α and INF-γ levels in heart tissue of mice with myocardial ischemia-reperfusion (Figure 3(h-o)). Besides, we validated that KLF16 could increase oxidative stress and inflammation in model of myocardial ischemia-reperfusion.

Serum of CK-MB and CK level (a and b), HE for myocardium fiber (c), left ventricular internal diameter (d), left ventricular ejection fraction (e), left ventricular fractional shortening (f) and Left ventricular stroke volume (g); MDA, SOD, GSH, GSH-PX levels in heart tissue (h, i, j and k); IL-1β, IL-6, TNF-α and INF-γ levels in heart tissue (l, m, n and o). Model, MI mice group; Model+anti-KLF16, MI mice with anti-KLF16 body group. n = 6; **p < 0.01 compared with MI mice group.

### KLF16 regulated inflammation in vitro model of myocardial ischemia-reperfusion

**Figure 4.**
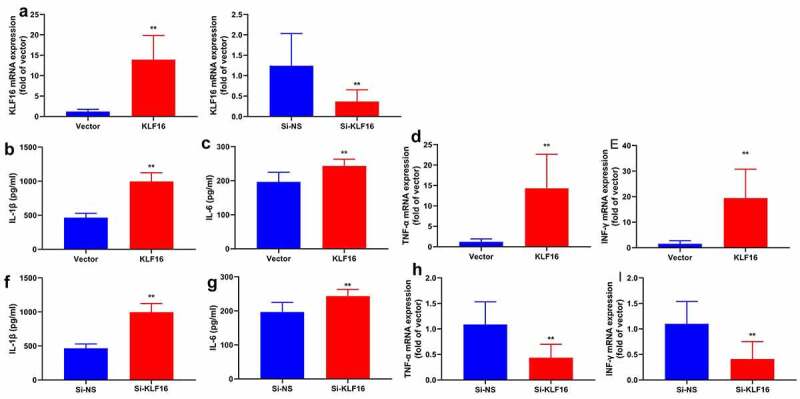
KLF16 regulated inflammation in vitro model of myocardial ischemia-reperfusion

Next, this study used KLF16 plasmid or si-KLF16 mimics to regulate KLF16 expression in vitro model of myocardial ischemia-reperfusion. KLF16 plasmid increased KLF16 expression in vitro model of myocardial ischemia-reperfusion (Figure 4(a)). At the same time, si-KLF16 mimics reduced KLF16 expression in vitro model of myocardial ischemia-reperfusion (Figure 4(a)). In addition, the over-expression of KLF16 reduced IL-1β, IL-6, TNF-α and INF-γ levels in vitro model of myocardial ischemia-reperfusion (Figures 4(b-e)). Meanwhile, the down-regulation of KLF16 increased promoted IL-1β, IL-6, TNF-α and INF-γ levels in vitro model of myocardial ischemia-reperfusion (Figures 4(f-i)). These results suggested that KLF16 is functionally relevant effector of regulating oxidative stress and inflammation in model of myocardial ischemia-reperfusion.

The expression mRNA of KLF16 (a) in vitro model; IL-1β (b), IL-6 (c), INF-γ (d) and TNF-α (e) in vitro model by over-expression of KLF16; IL-1β (f), IL-6 (g), INF-γ (h) and TNF-α (i) in vitro model by down-regulation of KLF16. Vector, negative control group; KLF16, over-expression of KLF16; Si-NS, Si-negative control group; Si-KLF16, down-regulation of KLF16 group. n = 3; **p < 0.01 compared with control group or Si-negative control group

### TFAM/PPARβ signal passage was target spot of KLF16 in myocardial ischemia-reperfusion

**Figure 5.**
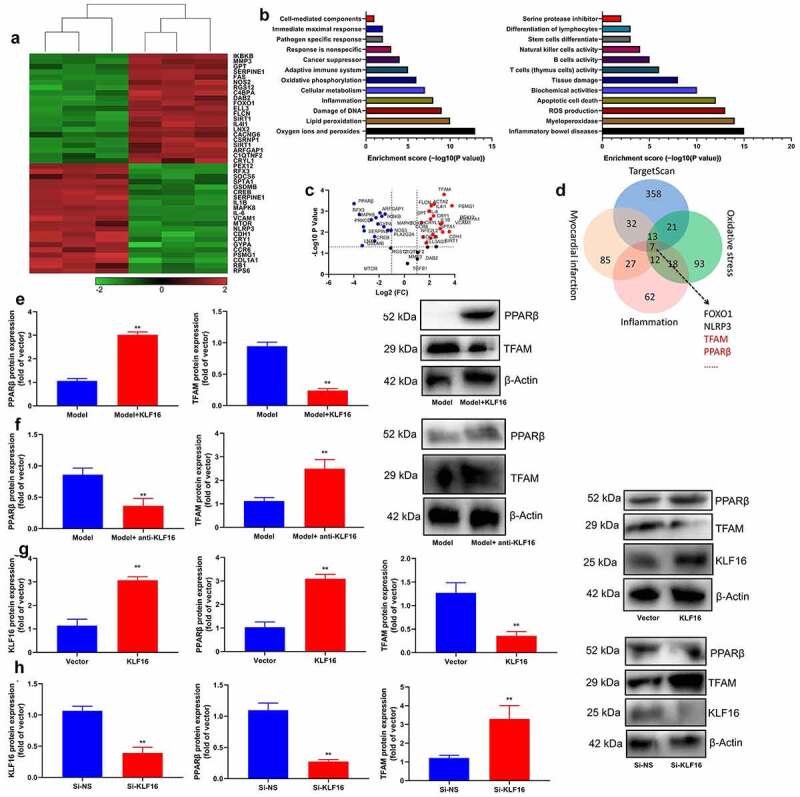
TFAM/PPARβ signal passage is target spot of KLF16 in Myocardial ischemia-reperfusion

In order to validate the mechanism of KLF16 in myocardial ischemia-reperfusion on oxidative stress and inflammation, we analyzed the gene expression levels of KLF16 in model of myocardial ischemia-reperfusion using microarray analysis (Figure 5(a,b)). As shown by these results of volcanic map and result analysis showed that, there was a significant difference between TFAM and PPARβ expression level. (Figure 5(c,d)). Human KLF16 recombinant protein induced PPARβ protein expression, and suppressed TFAM protein expression in heart tissue of mice with myocardial ischemia-reperfusion (Figure 5(e)). Anti-KLF16 body suppressed PPARβ protein expression and induced TFAM protein expression in heart tissue of mice with myocardial ischemia-reperfusion (Figure 5(f)). Meanwhile, over-expression of KLF16 not only induced PPARβ and KLF16 protein expressions, but also suppressed TFAM protein expression in vitro model of myocardial ischemia-reperfusion (Figure 5(g)). Apart from that, the down-regulation of KLF16 suppressed PPARβ and KLF16 protein expressions, and induced TFAM protein expression in vitro model of myocardial ischemia-reperfusion (Figure 5(h)).

Heat map (a), signal channel (b), volcanic map (c), result analysis chart (d); TFAM and PPARβ protein expressions in mice with KLF16 human recombinant protein group (e) (n = 6); TFAM and PPARβ protein expressions in mice with anti-KLF16 body group (f) (n = 6); KLF16, TFAM and PPARβ protein expressions in vitro model of over-expression of KLF16 group (g) (n = 3); KLF16, TFAM and PPARβ protein expressions in vitro model of over-expression of KLF16 group (h) (n = 3). Model, MI mice group; Model+KLF16, MI mice with KLF16 human recombinant protein group; MI mice with anti-KLF16 body group; Vector, negative control group; KLF16, over-expression of KLF16; Si-NS, Si-negative control group; Si-KLF16, down-regulation of KLF16 group.

**p < 0.01 compared with MI mice group, control group or Si-negative control group;

### TFAM interlink KLF16 in myocardial ischemia-reperfusion

**Figure 6.**
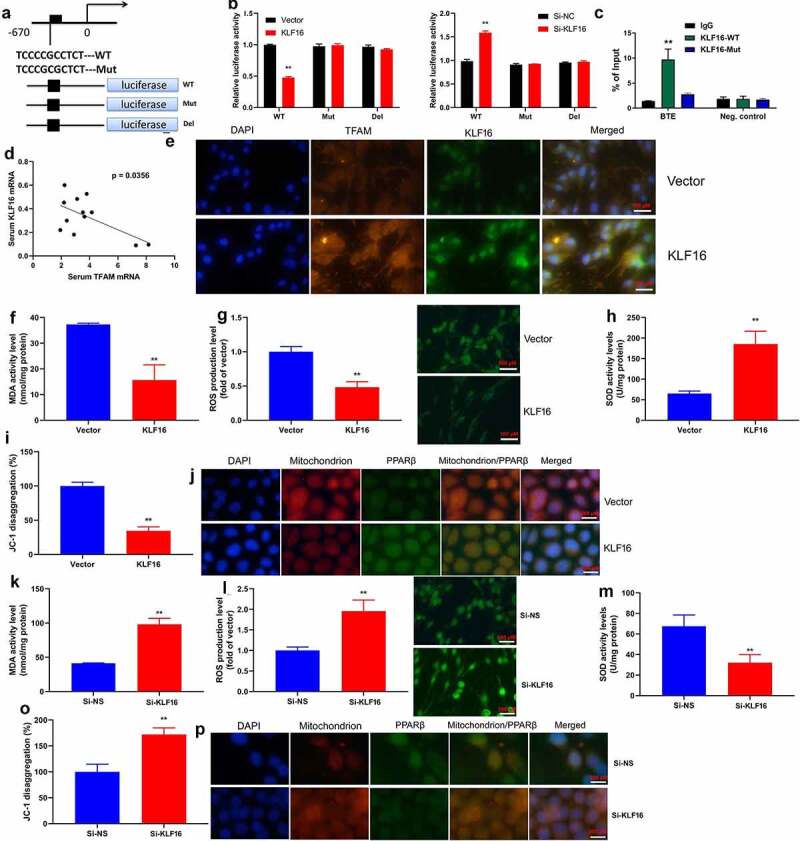
TFAM interlink KLF16 in Myocardial ischemia-reperfusion

The assessed the role of TFAM involved in the function of KLF16 in myocardial ischemia-reperfusion by interacting with its promoter. The TFAM promoter region was showed at Figure 6(a). As expected, over-expression of KLF16 significantly reduced the luciferase reporter activity level (Figure 6(b)). Additionally, the down-regulation of KLF16 increased luciferase reporter activity level (Figure 6(b)).ChIP assay reported that the high binding affinity of endogenous KLF16 to the BTE region in TFAM promoter (Figure 6(c)). Serum mRNA of KLF16 was negative correlation with serum TFAM mRNA expression in patients with myocardial ischemia-reperfusion (Figure 6(d)). Immunofluorescence showed that the over-expression of KLF16 suppressed TFAM expression in vitro model of myocardial ischemia-reperfusion (Figure 6(e)). Over-expression of KLF16 not only decreased MDA and ROS production levels, but also promoted SOD levels in vitro model of myocardial ischemia-reperfusion (Figures 6(f-h)). Additionally, over-expression of KLF16 reduced JC-1 disaggregation, increased mitochondrial damage and NLRP3 expression in vitro model of myocardial ischemia-reperfusion (Figure 6(i,j)). Down-regulation of KLF16 increased MDA and ROS production levels, and reduced SOD levels in vitro model of myocardial ischemia-reperfusion (Figure 6(k-m)). Down-regulation of KLF16 increased JC-1 disaggregation, and suppressed mitochondrial damage and NLRP3 expression in vitro model of myocardial ischemia-reperfusion (Figure 6(o,p)). Taken together, our results indicated that KLF16 suppressed TFAM expression, leading to oxidative stress and inflammation in model of myocardial ischemia-reperfusion.

TFAM promoter region (a), luciferase reporter activity (b), high binding affinity of endogenous KLF16 to the BTE region by ChIP assay (c), serum mRNA of KLF16 was negative correlation with serum TFAM mRNA expression in patients with myocardial ischemia-reperfusion (d); KLF16 and TFAM expression by immunofluorescence (e), MDA, ROS and SOD levels (F, G and H), JC-1 disaggregation (i), mitochondrial damage and NLRP3 expression by immunofluorescence (j) in vitro model of myocardial ischemia-reperfusion by over-expression of KLF16; MDA, ROS and SOD levels (k, l and m), JC-1 disaggregation (o), mitochondrial damage and NLRP3 expression by immunofluorescence (p) in vitro model of myocardial ischemia-reperfusion by down-regulation of KLF16. Vector, negative control group; KLF16, over-expression of KLF16; Si-NS, Si-negative control group; Si-KLF16, down-regulation of KLF16 group.

n = 3; **p < 0.01 compared with control group or Si-negative control group;

### TFAM participate in KLF16 affected myocardial ischemia-reperfusion

**Figure 7.**
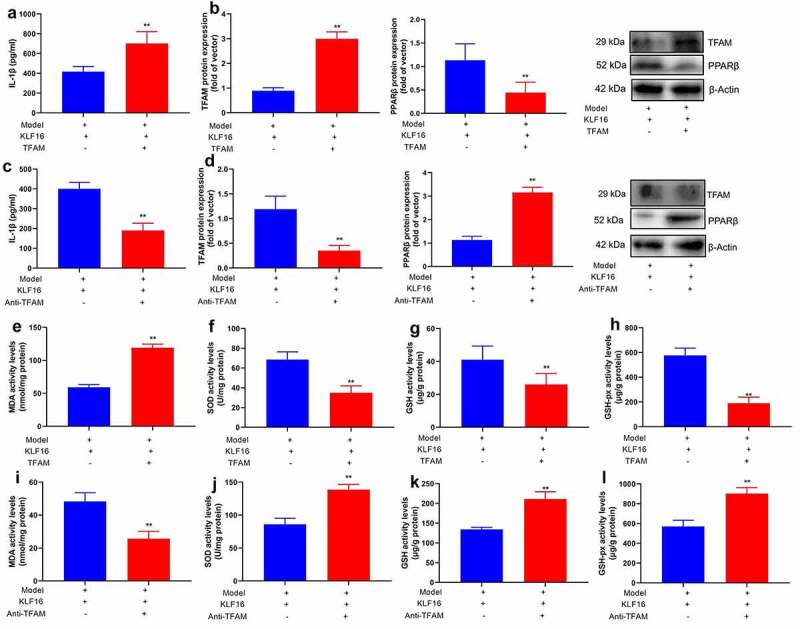
TFAM participate in KLF16 affects Myocardial ischemia-reperfusion in vivo model

The study further assessed that the role of TFAM in KLF16 affects myocardial ischemia-reperfusion. Human TFAM recombinant protein increased IL-1β level, induced TFAM protein expression, and suppressed PPARβ protein expression in mice of myocardial ischemia-reperfusion by KLF16 human recombinant protein (Figure 7(a,b)). As a result, the anti-TFAM body suppressed TFAM protein expression, and induced PPARβ protein expression in mice of myocardial ischemia-reperfusion by KLF16 human recombinant protein (Figure 7(c,d)). Human TFAM recombinant protein increased MDA level, and reduced SOD, GSH and GSH-PX levels in mice of myocardial ischemia-reperfusion by KLF16 human recombinant protein (Figure 7(e-h)). Anti-TFAM body reduced MDA level, and increased SOD, GSH and GSH-PX levels in mice of myocardial ischemia-reperfusion by KLF16 human recombinant protein (Figure 7(i-l)).

IL-1β level (a), TFAM and PPARβ protein expression (b) in mice of myocardial ischemia-reperfusion by KLF16 human recombinant protein and KLF16 human recombinant protein; IL-1β level (c), TFAM and PPARβ protein expression (d) in mice of myocardial ischemia-reperfusion by KLF16 human recombinant protein and anti-TFAM body; MDA (e), SOD (f), GSH (g) and GSH-PX (h) levels in mice of myocardial ischemia-reperfusion by KLF16 human recombinant protein and KLF16 human recombinant protein; MDA (i), SOD (j), GSH (k) and GSH-PX (l) levels in mice of myocardial ischemia-reperfusion by KLF16 human recombinant protein and anti-TFAM body. Model, MI mice group; Model+KLF16, MI mice with KLF16 human recombinant protein group; MI mice with anti-KLF16 body group; n = 6; **p < 0.01 compared with MI mice group.

**Figure 8.**
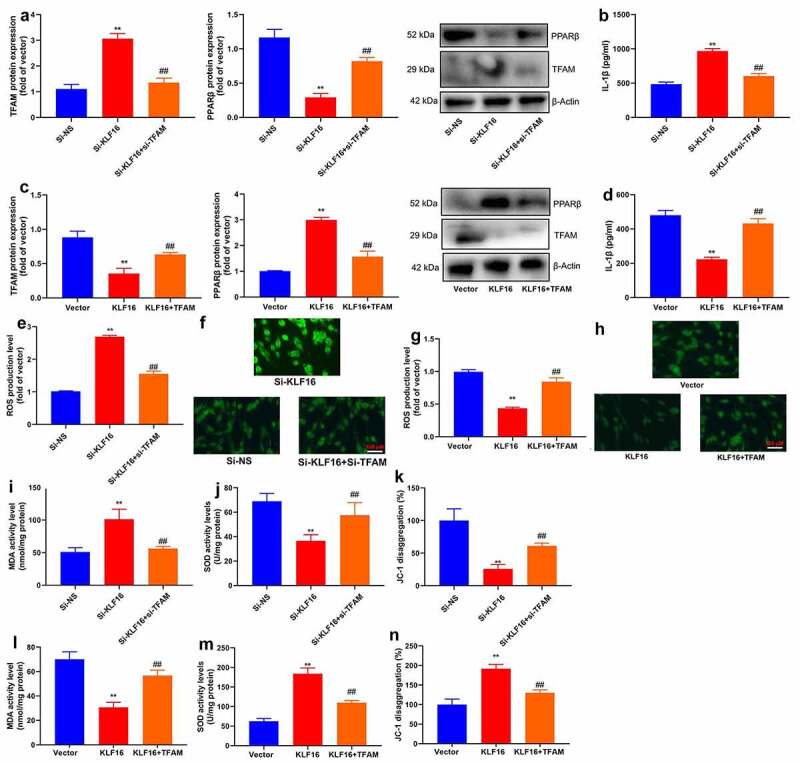
TFAM participate in KLF16 affects Myocardial ischemia-reperfusion in vitro model

For another,, si-TFAM not only suppressed the protein expression of TFAM and IL-1β level, but induced PPARβ protein expression in vitro model by down-regulation of KLF16 (Figure 8(a,b)). TFAM plasmid induced TFAM protein expression and IL-1β level, and suppressed PPARβ protein expression in vitro model by over-expression of KLF16 (Figure 8(c,d)).

TFAM and PPARβ protein expression (a), IL-1β level (b) in vitro model by down-regulation of TFAM and KLF16; TFAM and PPARβ protein expression (c), IL-1β level (d) in vitro model by over-expression of TFAM and KLF16;ROS production levels (e) in vitro model by down-regulation of TFAM and KLF16;ROS production levels (e) in vitro model by over-expression of TFAM and KLF16;MDA (I), SOD (J) and JC-1 disaggregation (k) in vitro model by down-regulation of TFAM and KLF16; MDA (l), SOD (m) and JC-1 disaggregation (n) in vitro model by over-expression of TFAM and KLF16. Vector, negative control group; KLF16, over-expression of KLF16 group; KLF16+ TFAM, over-expression of KLF16 and TFAM group; Si-NS, Si-negative control group; Si-KLF16, down-regulation of KLF16 group; Si-KLF16+ si-TFAM, down-regulation of KLF16 and TFAM group.n = 3; **p < 0.01 compared with control group or Si-negative control group; ##p < 0.01 compared with over-expression of KLF16 or down-regulation of KLF16 group.

Si-TFAM also reduced ROS production level in vitro model by down-regulation of KLF16 (Figure 8(e,f)). TFAM plasmid increased ROS production level in vitro model by up-regulation of KLF16 (Figure 8(g,h)).

Si-TFAM reduced MDA level, and increased SOD levels and JC-1 disaggregation in vitro model by down-regulation of KLF16 (Figure 8(i-k)). TFAM plasmid promoted MDA level, and decreased SOD levels and JC-1 disaggregation in vitro model by up-regulation of KLF16 (Figure 8(l-n)).

### PPARβ promoter region KLF16 affects myocardial ischemia-reperfusion

**Figure 9.**
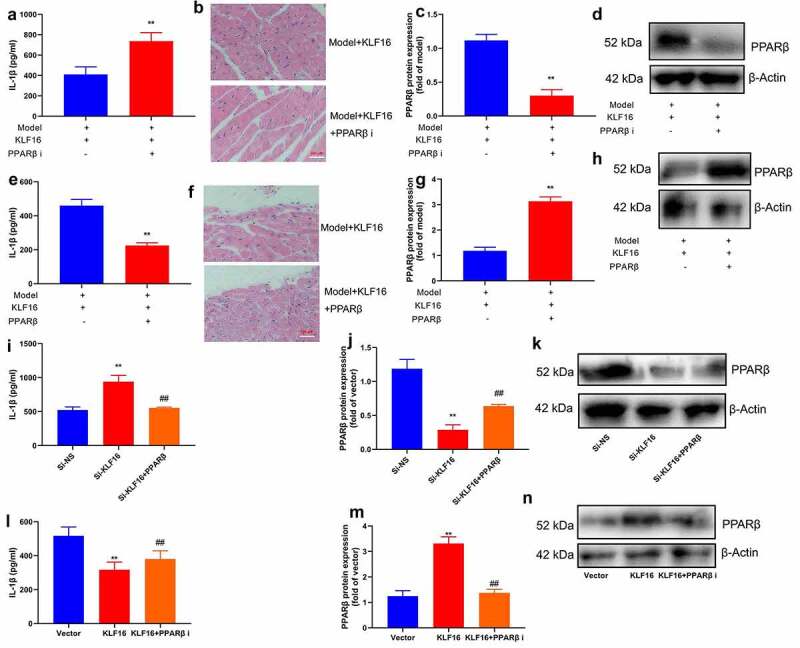
PPARβ promoter region KLF16 affects inflammation of myocardial ischemia-reperfusion

Furthermore,, PPARβ inhibitor (DG172 dihydrochloride, 1 mg/kg) increased IL-1β level, promoted myocardial damage, and suppressed PPARβ protein expression in mice of myocardial ischemia-reperfusion by KLF16 human recombinant protein (Figures 9(a-d)). PPARβ agonist (GW0742, 0.3 mg/kg) reduced IL-1β level, presented myocardial damage, as well as induced PPARβ protein expression in mice of myocardial ischemia-reperfusion by KLF16 human recombinant protein (Figure 9(e-h)). Notably, PPARβ agonist (GW0742, 1 μM) reduced IL-1β level, presented myocardial damage, and induced PPARβ protein expression in vitro model of myocardial ischemia-reperfusion by down-regulation of KLF16 (Figure 9(i-k)). Through up-regulation of KLF16, PPARβ inhibitor (DG172 dihydrochloride, 5 nM) increased IL-1β level, promoted myocardial damage, and suppressed PPARβ protein expression in mice of myocardial ischemia-reperfusion (Figure 9(l-n)).

IL-1β level (a), HE staining (b), PPARβ protein expression (c and d) in mice of myocardial ischemia-reperfusion by KLF16 human recombinant protein and PPARβ inhibitor; IL-1β level (e), HE staining (f), PPARβ protein expression (g and h) in mice of myocardial ischemia-reperfusion by KLF16 human recombinant protein and PPARβ agonist; IL-1β level (i), PPARβ protein expression (j and k) in vitro model by down-regulation of KLF16 and PPARβ agonist; IL-1β level (l), PPARβ protein expression (m and n) in vitro model by over-expression of KLF16 and PPARβ inhibitor. Vector, negative control group; KLF16, over-expression of KLF16; KLF16+ PPARβ i, over-expression of KLF16 and PPARβ inhibitor; Si-NS, Si-negative control group; Si-KLF16, down-regulation of KLF16 group; Si-KLF16+ PPARβ, down-regulation of KLF16 and PPARβ agonist group. n = 6; **p < 0.01 compared with control group or Si-negative control group; ##p < 0.01 compared with over-expression of KLF16 or down-regulation of KLF16 group.

**Figure 10.**
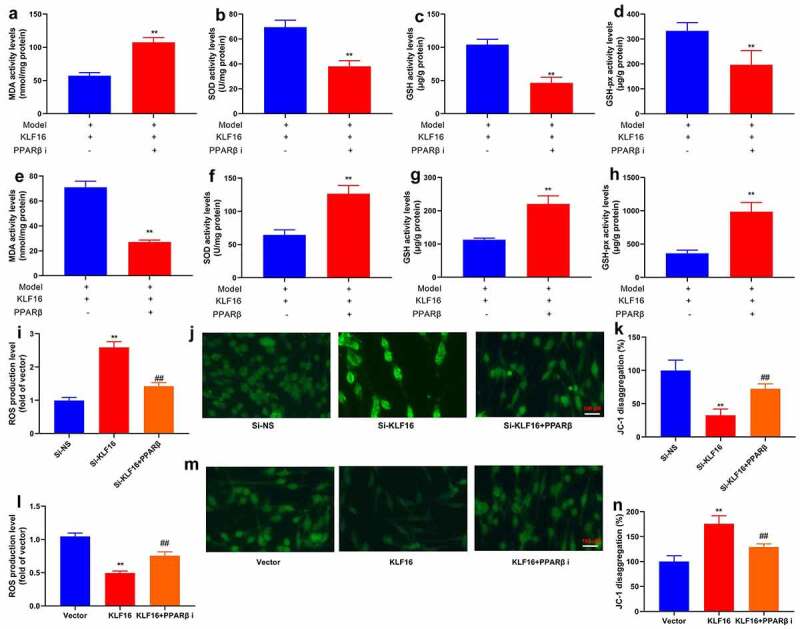
PPARβ promoter region KLF16 affects oxidative stress of myocardial ischemia-reperfusion

Interestingly, PPARβ inhibitor (DG172 dihydrochloride, 1 mg/kg) increased MDA level, and reduced SOD, GSH and GSH-PX levels in mice of myocardial ischemia-reperfusion by KLF16 human recombinant protein (Figure 10(a-d)). PPARβ agonist (GW0742, 0.3 mg/kg) reduced MDA level, and increased SOD, GSH and GSH-PX levels in mice of myocardial ischemia-reperfusion by KLF16 human recombinant protein (Figure 10(e-h)). Beyond that, PPARβ agonist (GW0742, 1 μM) reduced ROS production levels, and increased JC-1 disaggregation in vitro model of myocardial ischemia-reperfusion by down-regulation of Through up-regulation of KLF16, KLF16 (Figures 10(i-k)). PPARβ inhibitor (DG172 dihydrochloride, 5 nM) increased ROS production levels, and decreased JC-1 disaggregation in vitro model of myocardial ischemia-reperfusion (Figures 10(l-n)). Collectively, these data indicated that KLF16 reduced oxidative stress and inflammation to present myocardial ischemia-reperfusion by TFAM/PPARβ signal passage.

MDA (a), SOD (b), GSH (c) and GSH-PX (d) in heart tissue of mice with Myocardial ischemia-reperfusion by KLF16 human recombinant protein and PPARβ inhibitor; MDA (e), SOD (f), GSH (g) and GSH-PX (h) in heart tissue of mice with Myocardial ischemia-reperfusion by KLF16 human recombinant protein and PPARβ agonist; ROS production levels (i and j) and JC-1 disaggregation (k) in vitro model by down-regulation of KLF16 and PPARβ agonist; ROS production levels (l and m) and JC-1 disaggregation (n) in vitro model by over-expression of KLF16 and PPARβ inhibitor.Vector, negative control group; KLF16, over-expression of KLF16; KLF16+ PPARβ i, over-expression of KLF16 and PPARβ inhibitor;Si-NS, Si-negative control group; Si-KLF16, down-regulation of KLF16 group; Si-KLF16+ PPARβ, down-regulation of KLF16 and PPARβ agonist group.n = 3; **p < 0.01 compared with control group or Si-negative control group; ##p < 0.01 compared with over-expression of KLF16 or down-regulation of KLF16 group.

## Discussion

HF after MI is the most common type of chronic HF [3]. Recent studies have revealed that rapid and continuous activation of the inflammatory response is an important feature of HF after MI [3]. Appropriate inflammatory response can attenuate the scope of MI, promote scar formation and recovery of ischemic myocardium, while excessive and persistent activation of inflammatory response can promote heart enlargement, cardiac insufficiency and HF [3,8]. For the firstly time, we initially found that the expression of KLF16 mRNA and protein in patients or mice with myocardial ischemia-reperfusion were also increased. These results suggested that KLF16 may be participated in the pathogenesis and disease progression of myocardial ischemia-reperfusion.

When the content of mitochondrial ROS exceeds its internal antioxidant enzyme capacity, the excessive ROS is released into the cytoplasm, which in turn damages other organelles and cellular membrane structures, thereby causing myocardial damage [3]. In the present study, KLF16 reduced oxidative stress and inflammation, and presented MI in vivo model of myocardial ischemia-reperfusion. Sun et al. showed that KLF16 improve steatohepatitis and insulin resistance through regulation of ROS level [3]. These demonstrated that KLF16 had good oxidation and inflammation activity in myocardial ischemia-reperfusion.

Mitochondrial transcription factor A (TFAM) is an important factor involved in mitochondrial DNA transcription activation and regulation of mitochondrial DNA copy number [3]. TFAM is encoded by nuclear genes and translocated into mitochondria to play its regulatory roles [3]. Studies have shown that TFAM plays a role as a tumor-promoting gene and is involved in the tumorigenesis, development, invasion and metastasis of tumors [3]. TFAM is highly expressed in various human malignancies and is closely associated with the growth, invasion and metastasis of tumors [9]. The current study showed that TFAM interlink KLF16 in Myocardial ischemia-reperfusion, and KLF16 induced PPARβ protein expressions, and suppressed TFAM protein expression in vivo and vitro model of myocardial ischemia-reperfusion. Chen et al. showed that KLF16 suppresses cell proliferation of human glioma by targeting TFAM [3]. These results showed that KLF16 is related to inhibiting oxidative stress and inflammation in myocardial ischemia-reperfusion through the suppression of TFAM.

Oxidative stress and inflammation are involved in myocardial ischemia-reperfusion injury [10]. Myocardial cells produce a large amount of active ROS during reperfusion, and excessive ROS can induce a series of chemical reactions and cell damage, ultimately leading to cell dysfunction or apoptosis [3]. Recent studies have found that the activation of PPARβ has a protective effect on myocardial ischemia reperfusion injury [3]. Here, we showed TFAM/PPARβ signal passage is target spot of KLF16 in Myocardial ischemia-reperfusion. Sun et al. indicate that KLF16 improve steatohepatitis and insulin resistance by PPARα [3]. These results provide an evidence that KLF16/ TFAM/PPARβ pathway possibly mediates oxidative stress and inflammation in myocardial ischemia-reperfusion. We will research how TFAM regulates PPARβ in further experiment.

## Conclusion

In conclusion, the results from this study demonstrate that KLF16 reduced oxidative stress and inflammation, and presented MI in vivo model of myocardial ischemia-reperfusion through the induction of PPARβ by TFAM. KLF16 plays a key role in the maintenance of myocardial ischemia-reperfusion and KLF16 may alleviate oxidative stress and inflammation by activation of TFAM/PPARβ signaling pathway. Thus, this study suggests that KLF16 may provide a novel therapeutic strategy for myocardial ischemia-reperfusion.

## Highlights

TFAM interlink KLF16 in Myocardial ischemia-reperfusion.

knock-out KLF16 presented Myocardial ischemia-reperfusion.

knock-out KLF16 reduced oxidative stress and inflammation of myocardial ischemia-reperfusion.

KLF16 may provide a novel therapeutic strategy for myocardial ischemia-reperfusion.
